# Heterogeneity in postoperative intrinsic capacity trajectories in older adults with hip fractures: a prospective longitudinal study

**DOI:** 10.3389/fpubh.2026.1859779

**Published:** 2026-07-06

**Authors:** Wenya Qiang, Huolan Zhu, Chenni Dong, Xixi Zhang, Jiawei Fang, Qiaohui Ren, Zhijun Nie, Yulian Zhang

**Affiliations:** 1 Department of Nursing, Shaanxi Provincial People's Hospital, Xi'an, Shaanxi, China; 2 Department of Geriatrics, Shaanxi Provincial People's Hospital, Xi'an, Shaanxi, China; 3 Director's Office, Shaanxi Provincial People's Hospital, Xi'an, Shaanxi, China

**Keywords:** frailty, healthy aging, hip fracture, intrinsic capacity, recovery trajectory

## Abstract

**Objective:**

To investigate the group heterogeneity in postoperative intrinsic capacity (IC) recovery trajectories among older hip fracture (HF) patients and identify key independently associated factors.

**Methods:**

We conducted a prospective longitudinal study of 184 older patients following HF surgery at two tertiary hospitals. IC was assessed at 48 h, pre-discharge, 1 month, and 3 months post-surgery. A multi-state Markov model (MSM) was used to quantify transition intensity and probability between IC states, a latent class growth model (LCGM) was employed to identify heterogeneous trajectories of total IC scores, and predictors of trajectory categories were analyzed via multinomial logistic regression.

**Results:**

MSM revealed that patients in the “severe IC impairment” state had a 96.3% probability of remaining in that state throughout the 3-month follow-up. LCGM further identified three distinct recovery trajectories: the Higher-Level Growth Group (21.2%), the Medium-Level Improvement Group (36.9%), and the Lower-Level Stability Group (41.8%). Multivariate analysis indicated that age 60–69 years was significantly associated with increased odds of belonging to the Medium-Level Improvement Group (OR = 18.12, 95% CI: 4.22–77.86) and the Higher-Level Growth Group (OR = 3.33, 95% CI: 1.10–10.09) compared to the Lower-Level Stability Group. Normal plasma albumin was significantly associated with increased odds of belonging to the Medium-Level Improvement Group (OR = 7.07, 95% CI: 1.30–38.51).

**Conclusion:**

From a public health perspective, the identification of three distinct recovery trajectories provides an evidence-based framework for population-level risk stratification. The large subgroup (41.8%) with persistent severe impairment represents a high-risk population that may require targeted resource allocation for post-acute and long-term care. These findings support the development of tiered rehabilitation strategies at the health system level, aligning with the goals of healthy aging.

## 1 Introduction

Hip fractures (HF) are a severe geriatric event associated with poor functional outcomes and significant mortality, posing a growing challenge with population ageing (1, 2) Postoperative recovery is often complicated by secondary conditions like malnutrition and frailty, leading to sustained functional impairment (3–6). Current clinical prediction relies heavily on static indicators such as age, which fails to capture an individual’s holistic functional reserve and dynamic recovery potential (2).

The World Health Organisation’s (WHO) intrinsic capacity (IC) framework addresses this by integrating five core domains-vitality, locomotion, cognition, psychology, and sensory function—offering a multidimensional and dynamic approach to assessing health in older adults (7, 8). Unlike the frailty concept, which focuses on deficit accumulation, the IC framework emphasizes positive, person-centered functional reserves, making it particularly suitable for tracking recovery potential after surgery.

While impairments in specific domains are known HF risk factors (9, 10), evidence on IC as a comprehensive construct applied to post-HF recovery trajectories remains limited. Prior studies have advanced our understanding of recovery heterogeneity but have important gaps: they have focused solely on motor function without operationalizing the multidimensional WHO IC framework, or have been limited to short-term acute recovery (11–13). No prior study has combined macro-level trajectory identification with micro-level state transition modeling within the IC framework.

To address these gaps, we employed a complementary analytical framework combining latent class growth modeling (LCGM) and multi-state Markov modeling (MSM). While alternative methods exist—such as group-based trajectory modeling (GBTM) and growth mixture modeling (GMM)—each has limitations for our research question. LCGM balances parsimony with flexibility for moderate sample sizes, while MSM quantifies transition intensities between functional states. Their complementary value lies in distinct perspectives: LCGM identifies which patients follow which recovery pathways at the macro level; MSM quantifies how patients transition between functional states at the micro level.

Our specific objectives were to: (1) analyse dynamic transitions between IC states using MSM; (2) identify heterogeneous postoperative IC recovery trajectories using LCGM; and (3) determine key predictors of trajectory assignment—specifically age, albumin, and frailty—to provide evidence for early risk stratification and intensity-matched rehabilitation from a health system perspective. This evidence can inform population-level health management strategies, resource allocation, and the design of integrated care models that promote healthy aging.

## 2 Subjects and methods

### 2.1 Study design

This was a prospective longitudinal study designed to investigate the dynamic recovery trajectories of IC and their predictors in older patients undergoing HF surgery.

### 2.2 Study population

Between December 2024 and July 2025, older patients with HF undergoing surgery were recruited via convenience sampling from the orthopedic departments of two tertiary hospitals in Shaanxi Province, China. Inclusion criteria: (a) Age ≥60 years; (b) Imaging-confirmed primary, unilateral HF undergoing surgical treatment; (c) Alert consciousness with basic communication and literacy; (d) Informed consent and voluntary participation. Exclusion criteria: (a) Pathological fractures or concomitant fractures requiring surgery at other sites; (b) Pre-existing severe functional impairment (e.g., modified Rankin Scale score >4) (14) or severe dementia (Clinical Dementia Rating Scale ≥3) (15); (c) Expected survival <3 months; (d) Inability to complete follow-up.

Based on a sample size calculation for repeated-measures design (*α* = 0.05, power = 0.80, effect size *f* = 0.14, inter-measure correlation *r* = 0.5), a minimum of 142 participants was required. Ultimately, 184 patients completed all four follow-up assessments. For latent class growth models, simulation studies suggest that a sample size of approximately 150–200 is acceptable when class separation is good (entropy >0.9) and the smallest class proportion exceeds 20% (16). Our entropy (0.920) and smallest class proportion (21.2%) meet these criteria, supporting the adequacy of the sample size for trajectory analysis.

The study protocol was approved by the hospital ethics committee (Approval No. 2022K190), and written informed consent was obtained from all participants.

### 2.3 Data collection and measurement

#### 2.3.1 Follow-up time points

All assessments were conducted at four fixed time points: within 48 h post-surgery (T1), 1 day prior to discharge (T2), 1 month post-surgery (T3), and 3 months post-surgery (T4).

#### 2.3.2 Assessment of IC

IC was assessed using the WHO framework (17). Its five domains were evaluated with the following validated tools and scoring criteria: vitality by the Mini-Nutritional Assessment Short-Form (MNA-SF) (18), locomotion by the Short Physical Performance Battery (SPPB) (19), cognition by the Mini-Mental State Examination (MMSE) (20), psychological status by the 15-item Geriatric Depression Scale (GDS-15) (21), and sensory function by a self-report questionnaire. Following Gutiérrez-Robledo et al. (22) (who developed these cut-offs in a Mexican general aging cohort, as no validated cut-offs are yet available for Chinese surgical populations) raw scores from each tool were converted to a three-level domain score: 0 (severe impairment), 1 (mild-to-moderate impairment), or 2 (normal). The sum of the five domain scores yielded a total IC score ranging from 0 to 10, which was then categorized into three functional states: intact (9–10), mild-to-moderate impairment (6–8), and severe impairment (≤5). Detailed scoring cut-offs for each tool are provided in Table 1.

**Table 1:** Scoring criteria for each intrinsic capacity domain.

Domain	Assessment tool	Domain score		
		0 (severe)	1 (mild–moderate)	2 (normal)
Vitality	MNA-SF	≤7	8–11	≥12
Locomotion	SPPB	0–2	3–9	10–12
Cognition	MMSE	≤9	10–26	≥27
Psychology	GDS-15	10–15	5–9	0–4
Sensory	Self-report questionnaire	Both hearing and vision impaired	One impaired	Both normal

#### 2.3.3 Predictors and covariates

Variables for subsequent regression analyses were selected *a priori* based on clinical relevance. Frailty was assessed at baseline using the FRAIL scale, with a score ≥3 defining frailty (23). The final model included age, sex, plasma albumin, frailty status, number of comorbidities, and number of medication types.

### 2.4 Statistical methods

To identify heterogeneous developmental trajectories of IC, latent class growth models (LCGMs) were fitted using Mplus 8.3. Models with one to five classes were estimated sequentially. The optimal number of classes was determined based on the Akaike information criterion (AIC), Bayesian information criterion (BIC), adjusted BIC (aBIC), entropy (with values >0.8 indicating high classification accuracy), the Lo–Mendell–Rubin (LMR) test, and the bootstrap likelihood ratio test (BLRT). A significant LMR or BLRT (*p* < 0.05) indicated that a *k*-class model fitted better than a (*k*−1)-class model. Classes with a sample proportion <5% were considered unstable and thus not retained.

To analyze the dynamics of IC state changes, a continuous-time multi-state Markov model (MSM) was implemented using the msm package in R 4.0.0. Based on total IC scores, patients were classified into one of three transient states at each assessment: State 1 (Intact, 9–10 points), State 2 (Mild–Moderate impairment, 6–8 points), or State 3 (Severe impairment, ≤5 points). Transitions were restricted to adjacent states (i.e., between State 1↔2 and State 2↔3) because direct transition from Intact (State 1) to Severe impairment (State 3) within 3 months post-surgery is clinically implausible given the gradual nature of functional recovery after HF. This constraint follows the approach used in previous longitudinal studies of state transitions in geriatric conditions (24). The Markov assumption—that the transition intensity from one state to another depends only on the current state and not on the past history—was adopted as standard for multi-state models in panel data. The transition intensity matrix Q was estimated, from which the transition probability matrix P (t) was derived for each follow-up interval (T1–T2, T2–T3, T3–T4) to quantify state transition likelihoods.

To identify factors independently associated with trajectory class membership, multinomial logistic regression was performed using SPSS 27.0. The trajectory class (Higher-Level Growth, Medium-Level Improvement, or Lower-Level Stability) served as the dependent variable, with the Lower-Level Stability Group as the reference. Covariates were selected *a priori* based on clinical relevance and prior evidence, regardless of their univariate *p*-value. The final model included: age (60–69 years, 70–79 years, and ≥80 years, with ≥80 years as reference), sex, plasma albumin (normal vs. low), frailty status (FRAIL scale ≥3 vs. < 3), number of comorbidities, and number of medication types. All variables were entered simultaneously. A two-tailed *p* < 0.05 was considered statistically significant.

## 3 Results

### 3.1 Baseline characteristics of participants

A total of 201 patients were initially enrolled. During follow-up, 17 were lost (4 at T2, 7 at T3, 6 at T4), resulting in 184 patients (91.5%) who completed all assessments. Baseline characteristics did not differ significantly between completers and non-completers (all *p* > 0.05).

### 3.2 Postoperative trends in IC scores

**Figure 1:**
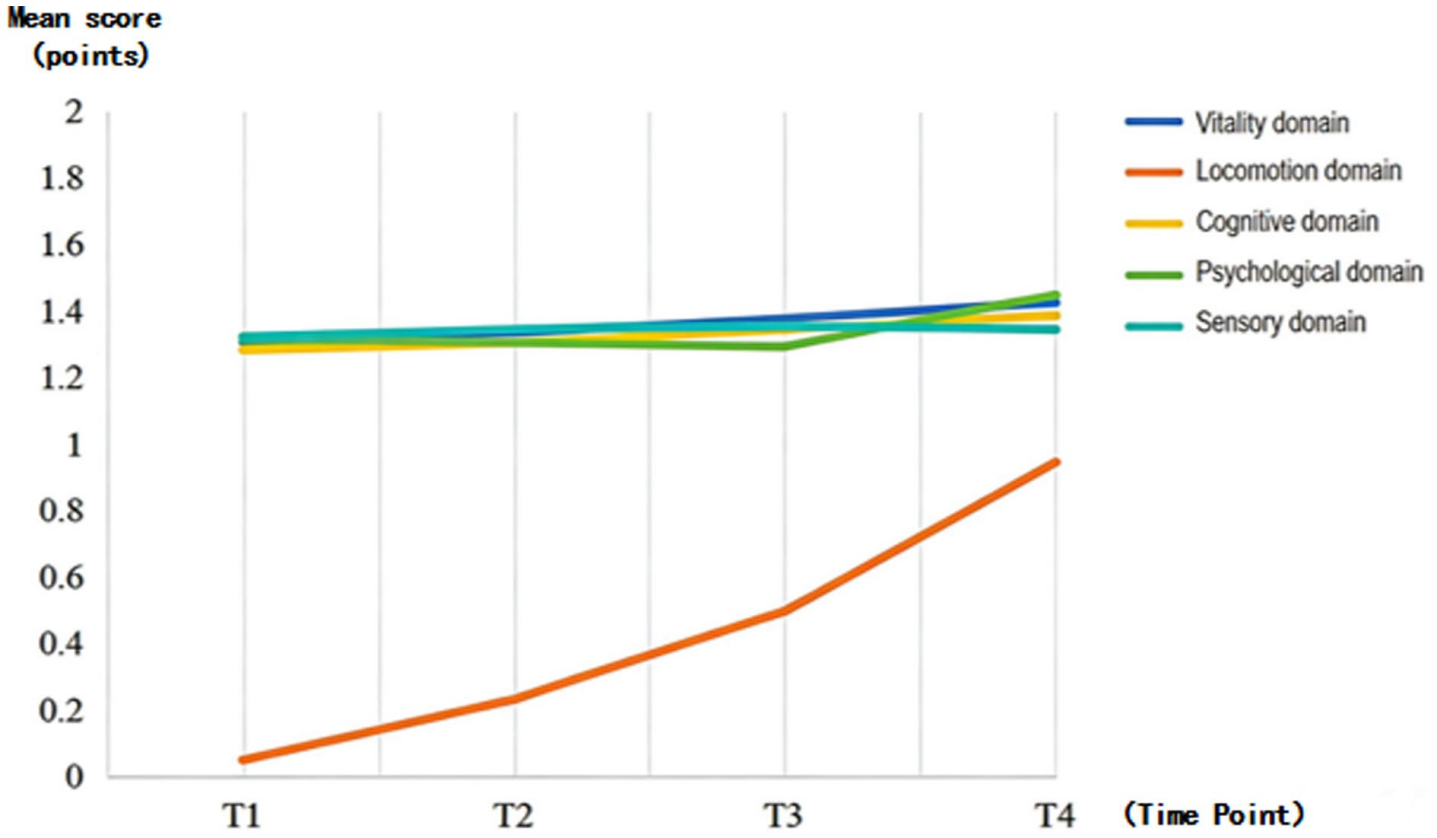
Trajectories of mean scores across the five IC domains during the 3-month postoperative period in older HF patients (*N* = 184). All dimensions changed significantly over time (*p* < 0.001).

The mean total IC score increased significantly from 5.27 at 48 h postoperatively (T1) to 6.52 at 3 months (T4) (*p* < 0.001). Analysis across the five IC domains revealed distinct recovery patterns (Figure 1). Scores in the vitality, locomotion, and cognitive domains showed sustained improvements throughout follow-up. Psychological scores exhibited a transient decline between discharge (T2) and 1 month (T3) before recovering, while sensory scores remained relatively stable.

### 3.3 Evolution of the proportion of functional status categories

**Figure 2:**
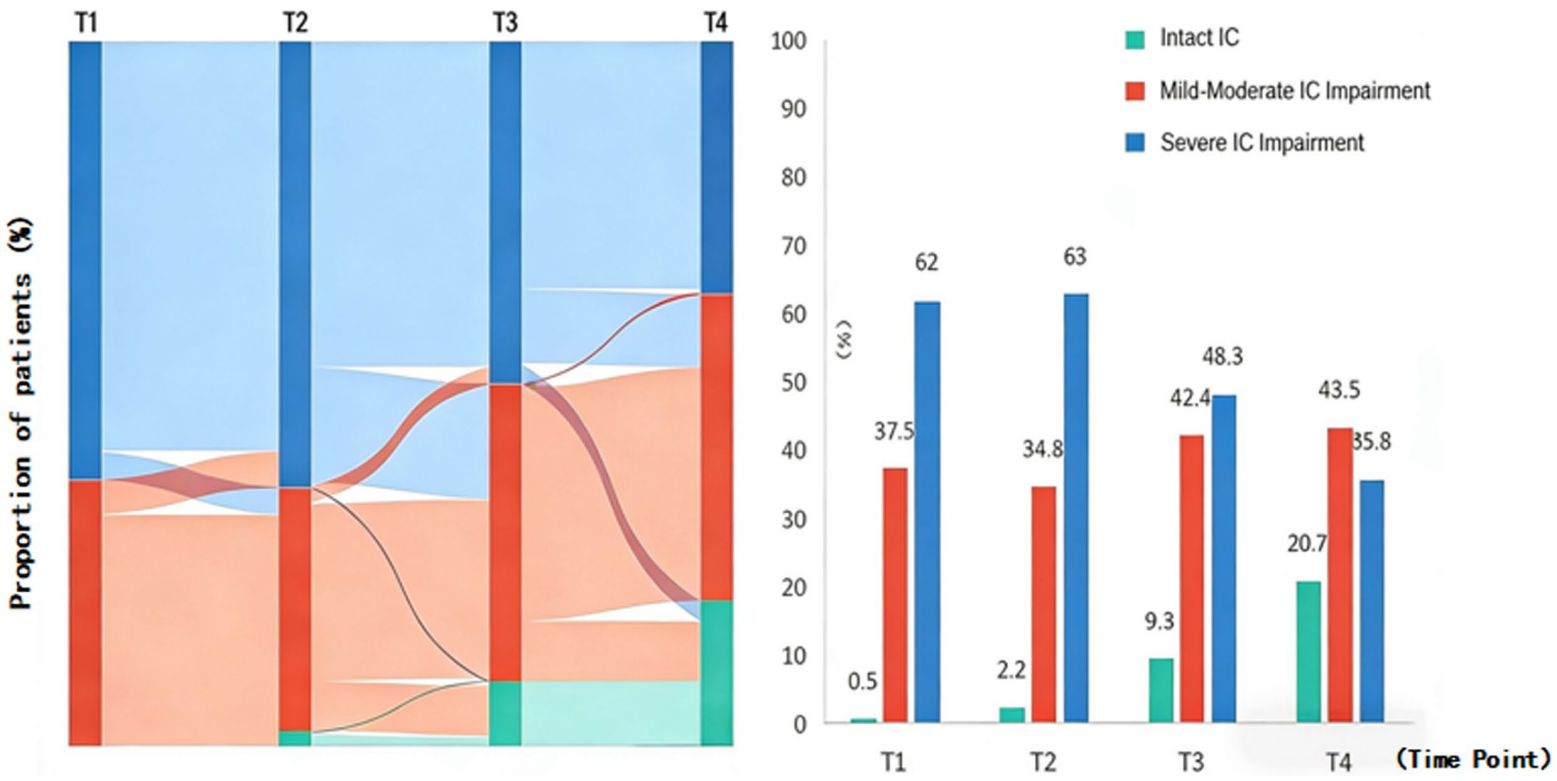
Transition pathways (left) and state proportions (right) of IC after HF surgery.

Figure 2 illustrates the sequential postoperative changes in patients’ IC status. From 48 h (T1) to 3 months (T4), the proportion of patients classified as having Severe IC Impairment decreased significantly from 62.0 to 35.8%, while those with Intact IC increased from 0.5 to 20.7%. Mild–Moderate IC Impairment emerged as the most prevalent state at T3 (42.4%) and T4 (43.5%). These shifts reflect a transition toward Intact IC and Mild–Moderate IC Impairment, with a corresponding impairment in Severe IC Impairment, indicating overall functional improvement.

### 3.4 Transition dynamics of postoperative IC states based on MSM

To elucidate the dynamic transitions underlying postoperative IC evolution, a continuous-time MSM was employed. This model classified patients into three ordered IC states at each assessment based on total scores: Intact IC (9–10 points), Mild–Moderate IC Impairment (6–8 points), and Severe IC Impairment (≤5 points), permitting transitions only between adjacent states.

**Figure 3:**
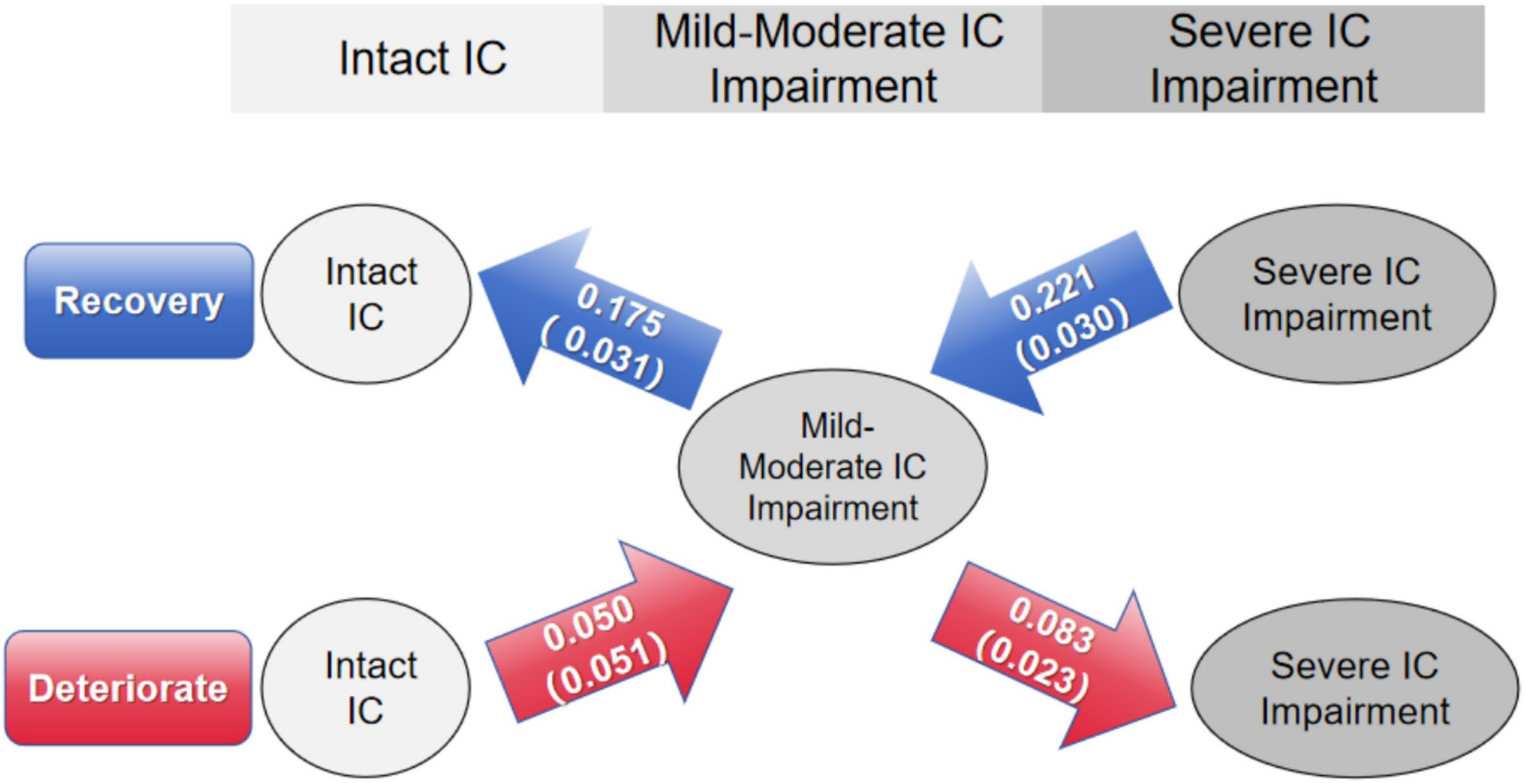
Transition intensities between postoperative IC states based on the MSM. Arrows denote permitted transitions between adjacent states: solid blue arrows represent improvement toward better states, dashed red arrows represent deterioration toward poorer states. Values on arrows indicate transition intensity estimates (standard errors in parentheses).

Analysis of transition intensities revealed a consistent directional tendency toward recovery (Figure 3). The intensity of improvement from Severe to Mild–Moderate Impairment (*q*₃₂ = 0.221, SE = 0.030, 95% CI: 0.162–0.280) was 2.66 times greater than the risk of deterioration in the opposite direction (*q*₂₃ = 0.083, SE = 0.023, 95% CI: 0.038–0.128). Similarly, the intensity from Mild–Moderate Impairment to Intact IC (*q*₂₁ = 0.175, SE = 0.031, 95% CI: 0.114–0.236) exceeded the reverse transition (*q*₁₂ = 0.050, SE = 0.051, 95% CI: −0.050–0.150).

**Figure 4:**
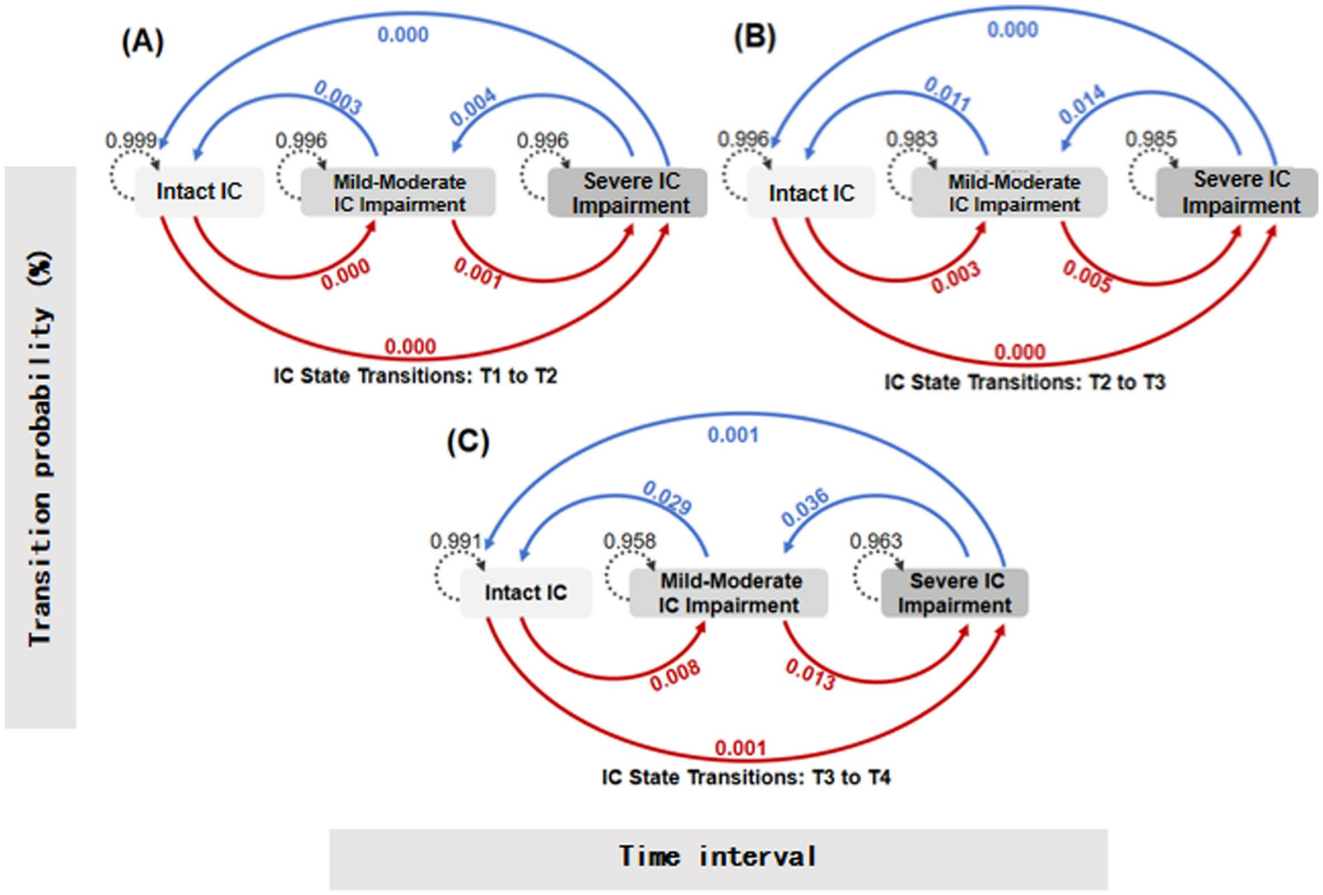
Transition probabilities between IC states over three consecutive postoperative intervals. **(A)** T1 to T2, **(B)** T2 to T3, **(C)** T3 to T4. Arrows represent permitted transitions: solid blue arrows indicate improvement toward better states, solid red arrows indicate deterioration toward poorer states, and dashed black arrows indicate remaining in the same state. Values on arrows denote conditional transition probabilities.

Despite this propensity for improvement, estimated transition probabilities indicated strong persistence in baseline IC states throughout follow-up (Figure 4). Notably, patients in Severe IC Impairment showed minimal recovery momentum, with a 96.3% probability of remaining in that state over 3 months and only a 3.6% chance of improving to a milder impairment level by the final interval. Patients in Mild–Moderate IC Impairment had a modest probability of improving to Intact IC (2.9% during T3–T4) and a low risk of deteriorating to Severe Impairment (1.3%). Conversely, Intact IC demonstrated high stability, with a 99.1% probability of being maintained at the end of follow-up.

### 3.5 Identification and determination of postoperative IC trajectories in older HF patients

LCGM was employed to conduct latent class analysis on the IC scores of older HF patients from 48 h post-surgery to 3 months post-surgery. This study extracted 1–5 latent classes to determine the optimal number for identifying IC development trajectories, with the fitted indices presented in Table 1.

As shown in Table 2, AIC, BIC, and aBIC values decreased with an increasing number of trajectory classes. All models exhibited entropy >0.8, indicating acceptable classification accuracy. The three-class model achieved an entropy of 0.920, approaching the ideal value of 1, reflecting excellent inter-class discrimination. Its LMR and BLRT results were both statistically significant (*p* < 0.05). In contrast, the LMR for the four-class model was non-significant, and its entropy was lower. Although the five-class model had the highest entropy and significant LMR/BLRT tests, it contained one class with only 2 patients (0.11%), which is clinically uninterpretable and likely reflects overfitting. Considering all fit indices, the three-class model was selected as optimal for representing postoperative IC trajectories.

**Table 2:** Model fit indices for LCGM of postoperative IC.

Category	AIC	BIC	aBIC	Entropy	LMR	BLRT	Category probability (%)
1	4470.026	4489.316	4470.312	–	–	–	100
2	4156.377	4185.312	4156.807	0.865	0.0000	0.0000	0.386/0.614
**3**	**4051.713**	**4090.293**	**4052.286**	**0.920**	**0.0035**	**0.0000**	**0.212/0.369/0.418**
4	4005.030	4053.254	4005.745	0.906	0.0640	0.0000	0.299/0.364/0.125/0.212
5	3986.561	4044.430	3987.420	0.927	0.0248	0.0000	0.212/0.364/0.294/0.120/0.011

① “–” indicates not applicable. ② The proportion of each subgroup was no less than 3%. Bold values indicate the optimal model selection. The three-class model was selected based on best model fit indices (lowest AIC, BIC, aBIC; highest entropy; significant LMR and BLRT; and clinically interpretable class proportions).

To further validate the three-class solution, we examined the average posterior probabilities for each class. The average posterior probabilities were 0.968 for C1, 0.974 for C2, and 0.971 for C3, all exceeding the conventional threshold of 0.70, indicating excellent classification accuracy (see Supplementary Table S1 for the full probability matrix). Therefore, the three-class model was retained as the most parsimonious and clinically meaningful solution.

**Figure 5:**
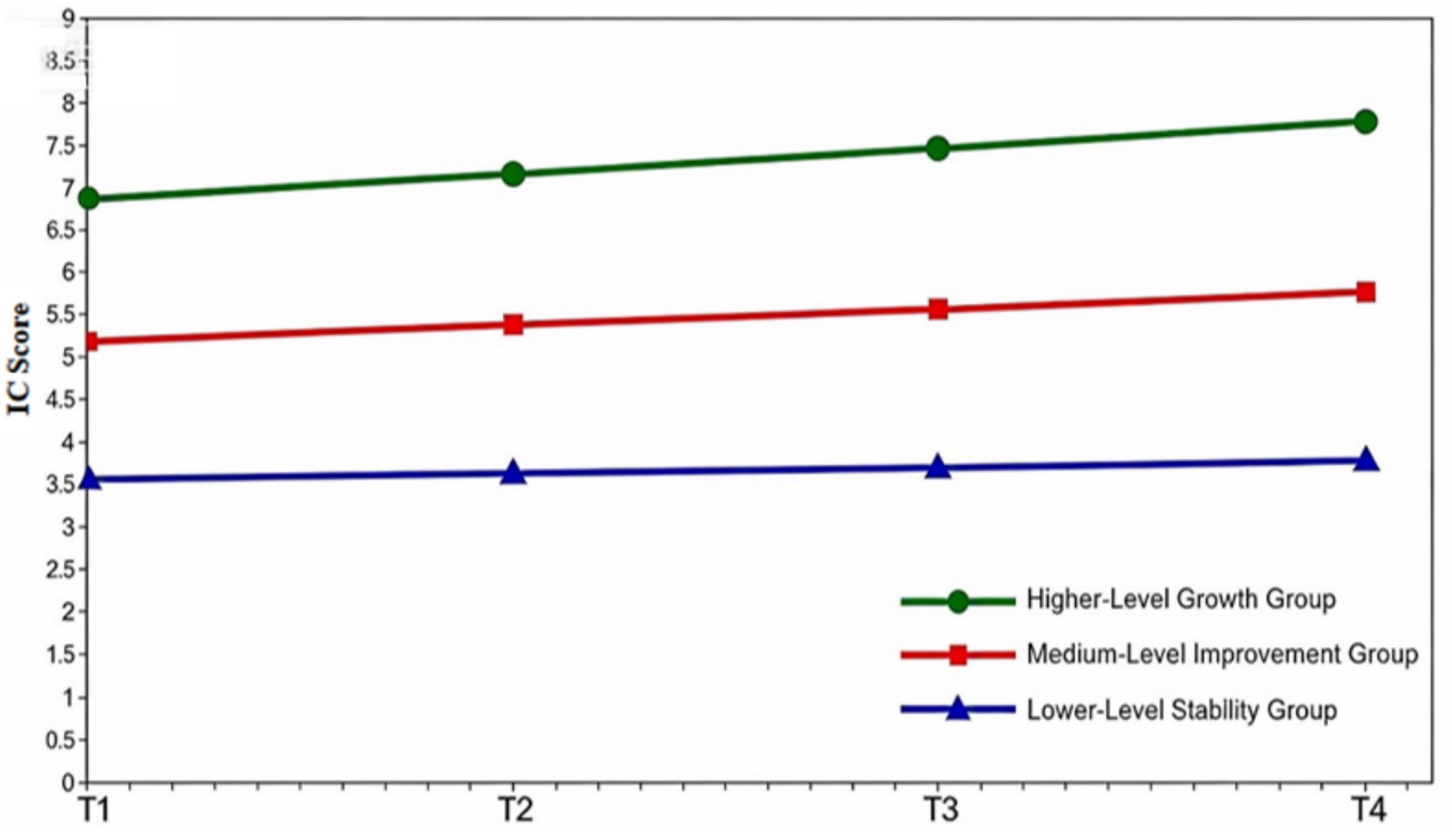
Latent class diagram of postoperative IC trajectories in older HF patients.

Based on LCGM, three distinct postoperative IC recovery trajectories were identified Figure 5. The Higher-Level Growth Group (C1, 21.2%, *n* = 39) was characterized by the highest baseline function (intercept = 6.869, *p* < 0.001) and the most rapid rate of improvement (slope = 0.292, *p* = 0.001). The medium-level improvement group (C2, 36.9%, *n* = 68) showed a moderate baseline (intercept = 5.182, *p* < 0.001) with a significant upward trajectory (slope = 0.182, *p* = 0.049). In contrast, the lower-level stability group (C3, 41.8%, *n* = 77) presented the lowest baseline (intercept = 3.541, *p* < 0.001) and exhibited no significant change over time (slope = 0.067, *p* = 0.381).

### 3.6 Factors associated with trajectory group membership

Multinomial logistic regression was performed with the Lower-Level Stability Group as the reference. Covariates were selected *a priori* based on clinical relevance and included age (categorical: 60–69 years, 70–79 years, and ≥80 years, with ≥80 years as reference), sex, plasma albumin (normal vs. low), frailty status, number of comorbidities, and number of medication types. As shown in Table 3, age 60–69 years was significantly associated with higher odds of belonging to the Medium-Level Improvement Group (OR = 18.12, 95% CI: 4.22–77.86) and the Higher-Level Growth Group (OR = 3.33, 95% CI: 1.10–10.09) compared to the Lower-Level Stability Group. Normal plasma albumin was also significantly associated with higher odds of belonging to the Medium-Level Improvement Group (OR = 7.07, 95% CI: 1.30–38.51). Frailty, sex, number of comorbidities, and number of medication types were not statistically significant in the final model (all *p* > 0.05).

**Table 3:** Multivariate logistic regression analysis of predictors of intrinsic capacity trajectories.

Variable	Group	*β*	SE	Wald *χ*^2^	*p*	OR	95%CI	
							Min	Max
C1 vs. C3^a^								
Age	60–69 years (vs. ≥ 80)	1.202	0.566	4.514	0.034	3.33	1.10	10.09
C2 vs. C3^a^								
Age	60–69 years (vs. ≥ 80)	2.897	0.744	15.172	<0.001	18.12	4.22	77.86
Plasma albumin	Normal (vs. low)	1.956	0.865	5.114	0.024	7.07	1.30	38.51

a C1 = Higher-Level Growth Group; C2 = Medium-Level Improvement Group; C3 = Lower-Level Stability Group. The model also adjusted for sex, number of comorbidities, number of medication types, and frailty, none of which were statistically significant (all *p* > 0.05). CI, confidence interval; OR, odds ratio.

## 4 Discussion

### 4.1 Dynamic patterns of postoperative transitions in IC status and their implications

The MSM shows that patients who are in a state of Severe IC Impairment immediately after surgery have a very high probability (96.3%) of remaining in that state over the three-month follow-up, and only a 3.6% chance of improving to Mild–Moderate Impairment. This suggests a recovery “plateau,” where conventional rehabilitation may be less effective for this subgroup. These patients, often with comorbidities and malnutrition, constitute a high-risk group for adverse outcomes such as disability and readmission (25). From a public health perspective, the identification of this high-risk subgroup has implications for health system planning. These patients are likely to require prolonged and resource-intensive care, placing potential demands on post-acute and long-term care services. Health systems should consider developing targeted programs for this population, such as transitional care units or community-based intensive rehabilitation, to prevent unnecessary hospital readmissions and institutionalization.

A closer analysis of transition dynamics reveals a critical paradox: although the intensity of improvement from Severe to Mild–Moderate IC Impairment (and from Mild–Moderate to Intact IC) exceeds the intensity of deterioration in the opposite directions, the actual probability of patients transitioning to a better state remains low. This highlights a gap between inherent recovery potential and its clinical realization, impeded by factors including frailty, inflammation, and malnutrition. This finding has dual implications: the greater intensity of improvement justifies proactive rehabilitation, yet the high persistence of Severe IC Impairment warns that conventional approaches may need to be supplemented for these patients. Consequently, a more integrated and intensive intervention system with progressive, phased goals may be needed (26).

Supporting this shift, evidence shows that multidimensional interventions targeting IC can maintain or enhance function in older adults with prior decline (27). Pertinently, a recent randomised controlled trial confirmed that multicomponent care-combining exercise, nutrition, and medication optimisation-significantly improved IC scores in community-dwelling older adults (28). Implementing similar integrated models for HF patients identified as being in a state of Severe IC Impairment offers a promising strategy to overcome functional recovery stagnation.

### 4.2 Heterogeneous patterns in postoperative IC recovery trajectories

LCGM revealed marked group heterogeneity in postoperative IC trajectories among older HF patients. This finding indicates that functional recovery follows diverse trajectories rather than a uniform pattern, providing preliminary evidence for future exploration of trajectory-based clinical stratification.

Patients in the Higher-Level Growth Group exhibited the highest baseline IC levels and the steepest improvement slope, indicating that they can draw upon superior physiological reserves to effectively counteract surgical stress and achieve optimal functional recovery. Notably, the significant improvement in the locomotion domain aligns with the core IC dimensions of the WHO framework (29). From a service planning perspective, this group may require only a streamlined rehabilitation pathway focused on maintaining gains and preventing deconditioning (30).

The Medium-Level Improvement Group (36.9%) presented with moderate initial IC scores and demonstrated a stable upward trajectory over time, reflecting considerable rehabilitative plasticity. For these patients, clinical interventions should actively engage their compensatory potential. Early screening and stratified management using IC assessment tools are essential, along with the development of personalized intervention plans that account for nutritional status, comorbidities, and motivation. In practice, this group is likely to benefit from moderately intensive, goal-oriented rehabilitation programs with regular monitoring to ensure they remain on an upward trajectory; any sign of deviation should trigger an immediate multidisciplinary review.

The Lower-Level Stability Group constituted the largest proportion (41.8%), exhibiting the lowest baseline function and no significant improvement during follow-up. This trajectory pattern corroborates the “sticky” state revealed by MSM, collectively indicating severely depleted physiological reserves and limited recovery potential within this cohort. Functional recovery in this group is likely constrained by depleted reserves and multi-system dysregulation, rendering conventional rehabilitation insufficient. Consequently, clinical strategies could consider addressing their unique recovery dynamics—characterized by low momentum and high resistance—through the early postoperative implementation of clearly defined, multi-targeted, and integrated interventions. Based on our observational findings, we speculate that given their high probability of remaining severely impaired, these patients may benefit from comprehensive geriatric assessment and multicomponent interventions (e.g., nutrition optimization, intensive physiotherapy, cognitive stimulation, and medication review) delivered in a coordinated manner, ideally within a transitional care unit or skilled nursing facility.

Despite the above clinical implications, we acknowledge that the three trajectories are distinguished primarily by their baseline IC levels rather than by distinct developmental patterns. Nevertheless, baseline IC level itself is clinically informative. The 3.33-point difference in mean baseline IC between the Lower-Level Stability and Higher-Level Growth Groups at 48 h post-surgery allows early identification of high-risk patients, supporting risk-stratified rehabilitation.

From a population health management perspective, the ability to stratify patients immediately post-surgery (48 h) using a simple baseline IC score has practical advantages. It does not require complex longitudinal data collection, making it feasible for routine clinical practice and large-scale implementation. Health systems could integrate this stratification into discharge planning protocols: patients in the Lower-Level Stability Group could be flagged for enhanced community support or transitional care, whereas those in the Higher-Level Growth Group might only need routine follow-up. This approach allows for more efficient allocation of limited rehabilitation resources, potentially reducing unnecessary healthcare utilization and costs while improving patient outcomes.

In summary, integrating the MSM with LCGM provides a dual perspective: macro-level heterogeneity in recovery trajectories and micro-level dynamics of state transitions. This underscores the need for future research to focus on developing targeted interventions that can increase the probability of transitioning to better functional states, thereby improving long-term recovery outcomes.

### 4.3 Key predictors of recovery trajectories

Age was the most consistent factor associated with better recovery trajectories. Younger age (60–69 years) was associated with higher odds of belonging to the more favourable trajectory groups. This finding aligns with the well-known decline in physiological reserves with advancing age, which limits functional restoration capacity after major surgery (31). From a clinical perspective, age-stratified rehabilitation strategies may be considered: younger patients might aim for functional maximization, while older patients may benefit from goals focused on functional maintenance and disability prevention.

Nutritional status, reflected by plasma albumin levels, was associated with moderate improvement. Normal albumin levels were linked to higher odds of belonging to the Medium-Level Improvement Group, but not to the highest-level recovery group after adjusting for other covariates. This suggests that good nutrition may support recovery, but achieving optimal recovery likely requires a combination of multiple factors beyond nutrition alone. Hypoalbuminaemia is known to reflect insufficient protein reserves and impaired repair capacity under surgical stress (32, 33), reinforcing the potential value of nutritional optimization in postoperative care.

Frailty, sex, comorbidity, and medication use were not statistically significant in the final model. However, this may be due to limited statistical power given the modest sample size, rather than a true absence of association, as frailty and multimorbidity are well-established adverse prognostic factors in hip fracture populations. Future larger studies should re-examine these factors.

Beyond physiological factors, recovery trajectories may also be influenced by social and behavioral factors. High levels of social engagement have been shown to reverse impaired IC and delay its decline (34). For patients with delayed recovery, integrating psychosocial activation strategies—such as group rehabilitation, family involvement, or digital platforms to maintain social connections—may help stimulate recovery motivation and overcome functional plateaus.

## 5 Conclusions and implications

### 5.1 Conclusion

This study identified three postoperative IC recovery trajectories among older hip fracture patients, providing a population-level framework for risk stratification. Age 60–69 years and normal plasma albumin were independently associated with more favourable trajectories. The large subgroup (41.8%) with persistent severe impairment represents a high-risk population that may require targeted health service planning and resource allocation. From a public health perspective, these findings support the development of tiered, population-based rehabilitation strategies to promote healthy aging and reduce long-term care burden. Future research should evaluate the cost-effectiveness and implementation feasibility of such stratified approaches in real-world health systems.

### 5.2 Limitations

This study has several limitations. First, the sample was recruited from two tertiary hospitals in a single province using convenience sampling, limiting generalizability. Second, the 3-month follow-up may not capture long-term recovery; the “Lower-Level Stability” label could represent delayed rather than permanent stagnation. Third, the sample size (*n* = 184) is modest for latent class modeling, and the smallest trajectory class (21.2%) may be unstable, which also precluded sensitivity analyses. Fourth, the MSM assumed adjacent-only transitions and time-homogeneous intensities; these assumptions were not formally tested due to sample size constraints. Fifth, 17 patients (8.5%) were lost to follow-up. Although baseline characteristics were comparable between completers and non-completers (Supplementary Table S2), complete-case analysis may introduce bias. Future multicentre studies with extended follow-up are needed to confirm our findings.

## Data Availability

The raw data supporting the conclusions of this article will be made available by the authors, without undue reservation.
