# Supplementary material for: Heterogeneity in postoperative intrinsic capacity trajectories in older adults with hip fractures: a prospective longitudinal study
Source: Front Public Health. 2026 Jul 6;14:1859779. doi: 10.3389/fpubh.2026.1859779 (PMC13381438; doi:10.3389/fpubh.2026.1859779)
Supplement: Supplementary file 1 [file Table_1.DOCX]

Supplementary Table S1. Average posterior probabilities for the three-class LCGM solution

|  | C1 | C2 | C3 |
| --- | --- | --- | --- |
| C1 | **0.968** | 0.000 | 0.026 |
| C2 | 0.000 | **0.974** | 0.000 |
| C3 | 0.053 | 0.000 | **0.971** |

The average posterior probabilities for the three latent classes are presented below. Values on the diagonal represent the probability of correct classification for each class. All diagonal values exceeded the conventional threshold of 0.70, indicating excellent classification accuracy.
