# Supplementary material for: Heterogeneity in postoperative intrinsic capacity trajectories in older adults with hip fractures: a prospective longitudinal study
Source: Front Public Health. 2026 Jul 6;14:1859779. doi: 10.3389/fpubh.2026.1859779 (PMC13381438; doi:10.3389/fpubh.2026.1859779)
Supplement: Supplementary file 2 [file Table_2.DOCX]

| Variable | Completers (n=184) | Non-completers (n=17) | P value |
| --- | --- | --- | --- |
| **Age, n (%)** |  |  | 0.572* |
| 60–69 years | 50 (27.2) | 4 (23.5) |  |
| 70–79 years | 57 (31.0) | 7 (41.2) |  |
| ≥80 years | 77 (41.8) | 6 (35.3) |  |
| **Sex, n (%)** |  |  | 0.492† |
| Male | 64 (34.8) | 6 (35.3) |  |
| Female | 120 (65.2) | 11 (64.7) |  |
| **Plasma albumin, n (%)** |  |  | 0.643† |
| Normal | 129 (70.1) | 8 (47.1) |  |
| Abnormal | 55 (29.9) | 9 (52.9) |  |
| **Number of comorbidities, n (%)** |  |  | 0.057* |
| ≤2 | 55 (29.9) | 8 (47.1) |  |
| 3–5 | 88 (47.8) | 9 (52.9) |  |
| >5 | 41 (22.3) | 0 (0.0) |  |
| **Number of medications, n (%)** |  |  | 0.081* |
| ≤2 | 62 (33.7) | 8 (47.1) |  |
| 3–5 | 82 (44.6) | 9 (52.9) |  |

Comparison of baseline characteristics between participants who completed follow-up (n=184) and those lost to follow-up (n=17).

Note: *Fisher's exact test; †Chi-square test. Abnormality definitions: plasma albumin normal range 35-55 g/L.*
